# Anticoagulants Influence the Performance of In Vitro Assays Intended for Characterization of Nanotechnology-Based Formulations

**DOI:** 10.3390/molecules23010012

**Published:** 2017-12-21

**Authors:** Edward Cedrone, Barry W. Neun, Jamie Rodriguez, Alison Vermilya, Jeffrey D. Clogston, Scott E. McNeil, Yechezkel Barenholz, Janos Szebeni, Marina A. Dobrovolskaia

**Affiliations:** 1 Nanotechnology Characterization Lab, Frederick National Laboratory for Cancer Research, Frederick, MD 21702, USA; edward.cedrone@nih.gov (E.C.); neunb@mail.nih.gov (B.W.N.); rodriguezjc@mail.nih.gov (J.R.); alison.vermilya@nih.gov (A.V.); clogstonj@mail.nih.gov (J.D.C.); mcneils@mail.nih.gov (S.E.M.); 2 Department of Biochemistry, Institute for Medical Research Israel-Canada, Hebrew University-Hadassah Medical School, P.O.B. 12272, Jerusalem 91120, Israel; chezyb@gmail.com; 3 Nanomedicine Research and Education Center, Institute of Pathophysiology, Semmelweis University Nagyvárad tér 4, 1089 Budapest, Hungary; jszebeni2@gmail.com; 4 SeroScience Ltd., Nagyvárad tér 4, 1089 Budapest, Hungary

**Keywords:** nanoparticles, liposomes, hemolysis, platelet aggregation, plasma coagulation, complement activation, leukocyte proliferation, cytokines, safety, immunotoxicity, in vitro

## Abstract

The preclinical safety assessment of novel nanotechnology-based drug products frequently relies on in vitro assays, especially during the early stages of product development, due to the limited quantities of nanomaterials available for such studies. The majority of immunological tests require donor blood. To enable such tests one has to prevent the blood from coagulating, which is usually achieved by the addition of an anticoagulant into blood collection tubes. Heparin, ethylene diamine tetraacetic acid (EDTA), and citrate are the most commonly used anticoagulants. Novel anticoagulants such as hirudin are also available but are not broadly used. Despite the notion that certain anticoagulants may influence assay performance, a systematic comparison between traditional and novel anticoagulants in the in vitro assays intended for immunological characterization of nanotechnology-based formulations is currently not available. We compared hirudin-anticoagulated blood with its traditional counterparts in the standardized immunological assay cascade, and found that the type of anticoagulant did not influence the performance of the hemolysis assay. However, hirudin was more optimal for the complement activation and leukocyte proliferation assays, while traditional anticoagulants citrate and heparin were more appropriate for the coagulation and cytokine secretion assays. The results also suggest that traditional immunological controls such as lipopolysaccharide (LPS ) are not reliable for understanding the role of anticoagulant in the assay performance. We observed differences in the test results between hirudin and traditional anticoagulant-prepared blood for nanomaterials at the time when no such effects were seen with traditional controls. It is, therefore, important to recognize the advantages and limitations of each anticoagulant and consider individual nanoparticles on a case-by-case basis.

## 1. Introduction

**Figure 1:**
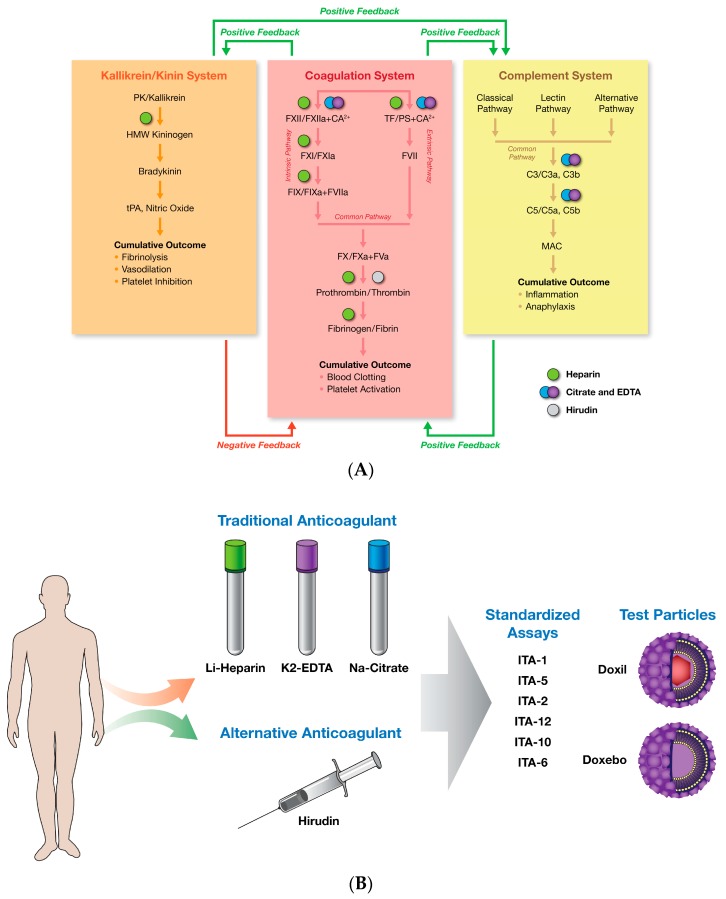
Rationale and study design. (**A**) Anticoagulants and their effects on various components of complement, plasma coagulation, and kinin/kallikrein systems are shown in this diagram. There is a cross-talk between these systems. Activation of the coagulation provides positive (i.e., agonist) feedback to the complement and kinin/kallikrein systems. Likewise, activated complement system stimulates coagulation, while activation of kinin/kallikrein system can also activate complement system. Cumulative outcome of the kallikrein/kinin system supplies a negative (i.e., inhibitory) feedback to the coagulation system to stop the blood clotting process and maintain hemostasis. Colored dots representing specific anticoagulant are shown above their respective protein targets. Unlike heparin, citrate, and EDTA, hirudin has a single target affecting only the coagulation cascade; (**B**) Schematic depiction of the study design. F—factor; C—complement; ITA—immunotoxicity assay from the standardized assay cascade (https://ncl.cancer.gov/resources/assay-cascade-protocols); TF—tissue factor; PS—phosphatidylserine; EDTA—ethylene diamine tetraacetic acid; PK—prekallikrein; tPA—tissue plasminogen activator; HMW—high molecular weight; MAC—membrane attack complex; K2—potassium ions.

Blood coagulates within minutes after collection. The process can be accelerated by environmental factors, such as temperature, which creates logistical challenges for researchers using the blood ex vivo to support pre-clinical safety studies of novel drug products, as well as for clinical blood tests [1]. This barrier can be overcome by adding various chemicals that prevent coagulation to the blood collection tubes [2]. Commonly used anticoagulants are ethylene diamine tetraacetic acid (EDTA), heparin, and citrate [2] (Figure 1A). Tubes of various sizes prefilled with one of these anticoagulants are available commercially and are widely used for a variety of in vitro assays as well as for ex vivo analysis of blood collected from patients. It is well established that each of these traditional anticoagulants has its own mechanism of action (MOA) (Figure 1A) [3]. For example, heparin, commonly used for cell-based and hemolysis assays, inhibits intrinsic, extrinsic, and common coagulation pathways by affecting several coagulation factors (FXII, FXI, FIX, Tissue Factor, thrombin, and fibrinogen). EDTA and citrate anticoagulants, commonly used in hematology tests, work by chelating divalent cations such as Ca^2+^ and Mg^2+^ required for both intrinsic and extrinsic pathways [3]. The MOA of EDTA and citrate anti-coagulants is reversible and can be overcome by supplementing an excess of divalent cations back into the blood. In addition to the coagulation system, all traditional anticoagulants also have targets in kinin/kallikrein and complement systems, which have close interactions with the coagulation system. Notably, heparin inhibits high-molecular-weight kininogen, while citrate and EDTA inhibit C3 and C5 convertases [3]. Interaction with multiple targets decreases the specificity of the traditional anticoagulants and creates limitations for their in vitro and in vivo use. Positive (activation) and negative (inhibition) feedback between coagulation, complement and kinin/kallikrein systems create additional complexities in the matrices prepared using traditional anticoagulants. The choice of anticoagulant for the in vitro and ex vivo assays is commonly driven by considerations of the assay type and the anticoagulant MOA due to the general recognition that the type of anticoagulant may influence optimal assay performance. Recently, a novel anticoagulant, hirudin, was discovered and proposed for in vitro and ex vivo blood-based assays due to its MOA specific to a single target, thrombin, in the common coagulation pathway. Several studies reported that hirudin is gentle to blood cells, minimally affecting their biochemical components, and suggested that it is a universal reagent that may overcome the need for the differential use of anticoagulants for various blood tests [3,4,5,6,7,8,9,10,11,12]. Hirudin is a protein present in the saliva of medicinal leeches [13,14,15] that was originally discovered by John Berry Haycraft in 1884, though it was only isolated in pure form in 1950. The structure was then determined in 1976. Natural hirudin is made of 65 amino acids and contains many isoforms [13,14,15]. There are several anti-coagulant formulations based on hirudin. One example is Desirudin, a recombinant, 65-amino-acid protein representing the hirudin variant 1 (HV1) isoform of hirudin that is used for deep vein thrombosis (DVT) prevention. Another formulation, Bivalirudin (also known as Angiomax and Angiox), is a synthetic, 20-amino-acid form of hirudin used for percutaneous coronary interventions [13,14,15].

The preclinical safety assessment of novel nanotechnology-based drug products frequently relies on in vitro assays, especially during the early stages of product development, due to the limited quantities of nanomaterials available for such studies. An in vitro assay cascade for the immunological safety assessment of nanomaterials and nanotechnology-formulated drugs and devices was developed in our lab in 2005 [16]. The assay cascade has been standardized and validated across 400 various nanotechnology platforms including, but not limited to, liposomes, emulsions, lipid nanoparticles, colloidal metals and metal oxides, carbon nanotubes, fullerenes, and polymeric nanomaterials. The purpose of this study was to compare the performance of several assays from this cascade when either traditional anticoagulants or hirudin were used to prevent coagulation of the healthy donor blood used for these assays. A well-characterized, clinically relevant PEGylated liposomal formulation of doxorubicin (Doxil) and its carrier Doxebo were used as model nanoparticles in this study. The study was performed as outlined in Figure 1B.

## 2. Results and Discussion

### 2.1. Physicochemical Characterization and Endotoxin

Blank and doxorubicin-loaded PEGylated liposomes (Doxebo and Doxil, respectively) were used as model particles in this study due to the wealth of information available about the platform and the drug from both preclinical and clinical experience (reviewed in [17,18]). The particles from the batch used in our study were analyzed by dynamic light scattering to verify the particle hydrodynamic size and zeta potential. The data confirmed that the basic physicochemical characteristics of both particles met the expectations for these nanomaterials (Table 1). Endotoxin, a common biological contaminant in nanoparticle formulations, may result in a false-positive response in immunological assays [19]. The hematology assays used in our study were not affected even by high endotoxin levels and can tolerate up to 500 EU/mL of the final endotoxin concentration [19,20,21]. However, leukocyte proliferation and cytokine assays are sensitive to as low as 1 EU/mL [19]. Since these assays require a 10-fold minimum required dilution (MRD), we set up a specification for the test nanoparticles to contain no more than 0.5 EU/mL of endotoxin. This level would mean that, even when the stock nanoparticle was used in the assay, after the 10-fold MRD, the final concentration of endotoxin in the culture would be less than 0.05 EU/mL, which is 20 times below the threshold concentration capable of inducing a false-positive response in the in vitro cytokine assay. Both Doxil and Doxebo samples comply with this specification (Table 1).

### 2.2. Hemolysis

**Figure 2:**
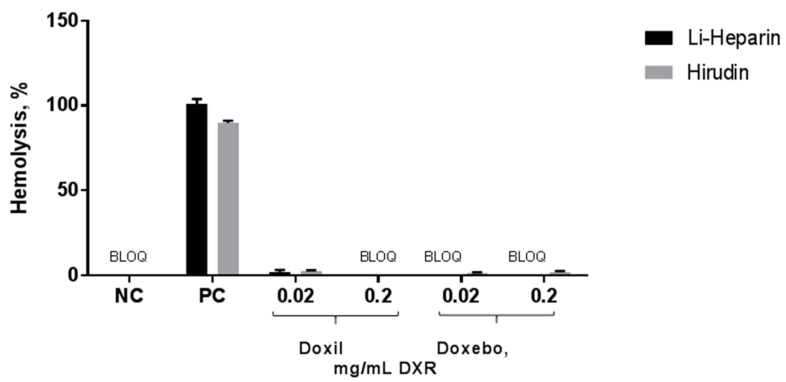
Hemolysis assay. Various, clinically relevant concentrations of Doxil and Doxebo were tested in the hemolysis assay in order to estimate their potential effects on the integrity of red blood cells. Three independent samples were prepared for each nanoparticle concentration and analyzed in duplicate (%CV < 20). Shown is mean (*n* = 3) ± SD. Triton X-100 was used as a positive control (PC). PBS was used as the negative control (NC). BLOQ: Below limit of quantification.

The hemolysis assay is commonly used to evaluate the effects of a test nanomaterial on the integrity of the red blood cells. As expected, the assay positive control resulted in nearly 100% lysis of the erythrocytes, while neither Doxebo nor Doxil were hemolytic. There was no difference between lithium-heparin- and hirudin-anticoagulated blood in this assay (Figure 2).

### 2.3. Platelet Aggregation

**Figure 3:**
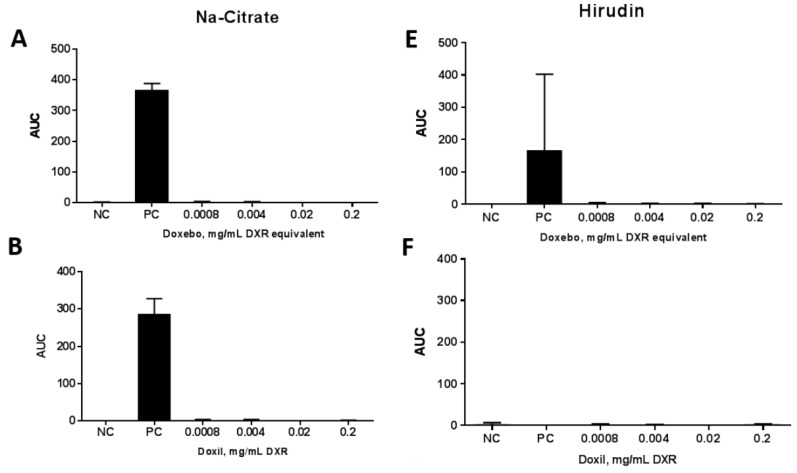

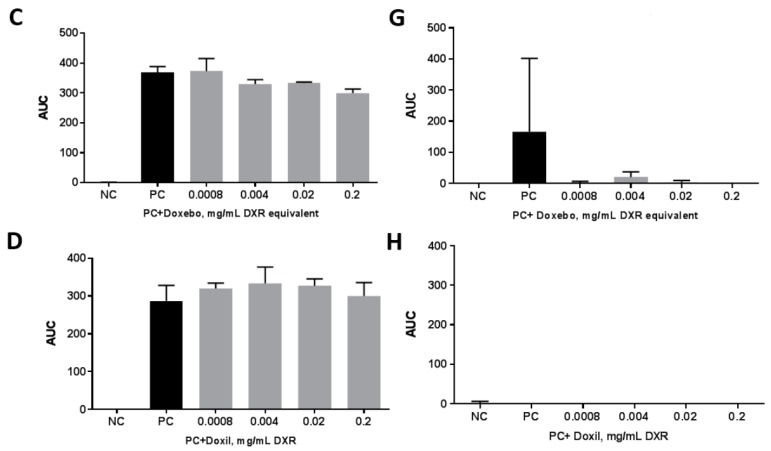
Platelet aggregation assay. Various clinically relevant concentrations of Doxil and Doxebo were spiked into platelet reach plasma (PRP), and platelet aggregation was monitored in real time during six minutes of sample incubation at 37 °C (**A**,**B**,**E**,**F**). Particle effects on collagen-induced platelet aggregation were tested by adding the collagen into PRP spiked with test nanomaterials (**C**,**D**,**G**,**H**). The AUC of nanoparticle-treated plasma (**A**,**B**,**E**,**F**) were compared to the negative control sample (NC). The AUC of the collagen-treated plasma pre-incubated with nanoparticles (**C**,**D**,**G**,**H**) was compared to the AUC of the PC. PC was collagen. NC was PBS. Plasma anticoagulated with Na-citrate (**A**–**D**) was compared to plasma anticoagulated with hirudin (**E**–**H**). Blood from the same donor volunteers was used for all tests. Doxebo and Doxil samples were conducted on different days due to the low throughput of the light transmission aggregometry. Shown is mean ± SD (*n* = 3).

A platelet aggregation assay is commonly used to answer two questions: is the test particle pro-thrombogenic, and does it have anti-platelet activity? First, we tested whether a given nanomaterial was capable of activating the platelets and inducing their aggregation. When the platelet-rich plasma (PRP) is spiked with a positive control collagen, platelets aggregate. Such aggregation increases light transmission, which is recorded by the light transmission aggregometer to generate the area under the curve (AUC). The higher the AUC, the greater the ability of the test sample to induce platelet aggregation. Second, we tested whether a given nanomaterial was capable of affecting platelet aggregation induced by a known aggregation stimulus. In this case, the PRP was pre-incubated with test nanomaterials and then spiked with collagen. If the test nanomaterial had a property of inhibiting platelet aggregation, the AUC, in response to the collagen in the nanoparticle pre-treated PRP, decreased in comparison to the control plasma spiked only with collagen. Sodium citrate is traditionally used to anticoagulate the donor blood for this test. When the assay was conducted in the citrated PRP, we observed an AUC in the collagen-treated sample within the expected range of approximately 300 (Figure 3A–D). Neither Doxebo nor Doxil induced platelet aggregation (Figure 3A,B, respectively) or inhibited collagen-induced platelet aggregation (Figure 3C,D, respectively). These results were expected based on the current knowledge about these particles. In contrast, when hirudin-anticoagulated blood was used to prepare PRP, the assay performance was disturbed (Figure 3E–H). The AUC in the positive control sample was completely suppressed or significantly lower than the AUC in the positive control sample generated using citrated PRP (Figure 3, compare positive controls (PC) between panels A–D and E–H). This data demonstrates that hirudin is suboptimal for the in vitro platelet aggregation assays. Our experience is different from that reported by Wallen et al., who did not detect a difference in collagen-induced platelet aggregation between heparin and hirudin anticoagulated blood [2]. The explanation for the discrepancy in the test results may come from the concentration of the hirudin, which was 20 µg/mL and 45 µg/mL, respectively. Our findings are in agreement with the study by Engstad et al., which reported that activated platelets isolated from hirudin-anticoagulated blood produced lower levels of platelet factor 4 than cells isolated from blood anticoagulated with traditional anticoagulants [22]. The concentration of hirudin used in the study by Engstad et al., was 10 µg/mL. Recombinant hirudin was used in both our study and the study by Engstad et al. [22], while Wallent et al., used desulphatohirudin [2]. These data suggest that hirudin’s effects on platelet activation may depend on both the concentration and type of hirudin. We speculate that the presence of unidentified impurities in the hirudin preparations used in all studies may further contribute to the discrepancy in the test results.

### 2.4. Plasma Coagulation Time

**Figure 4:**
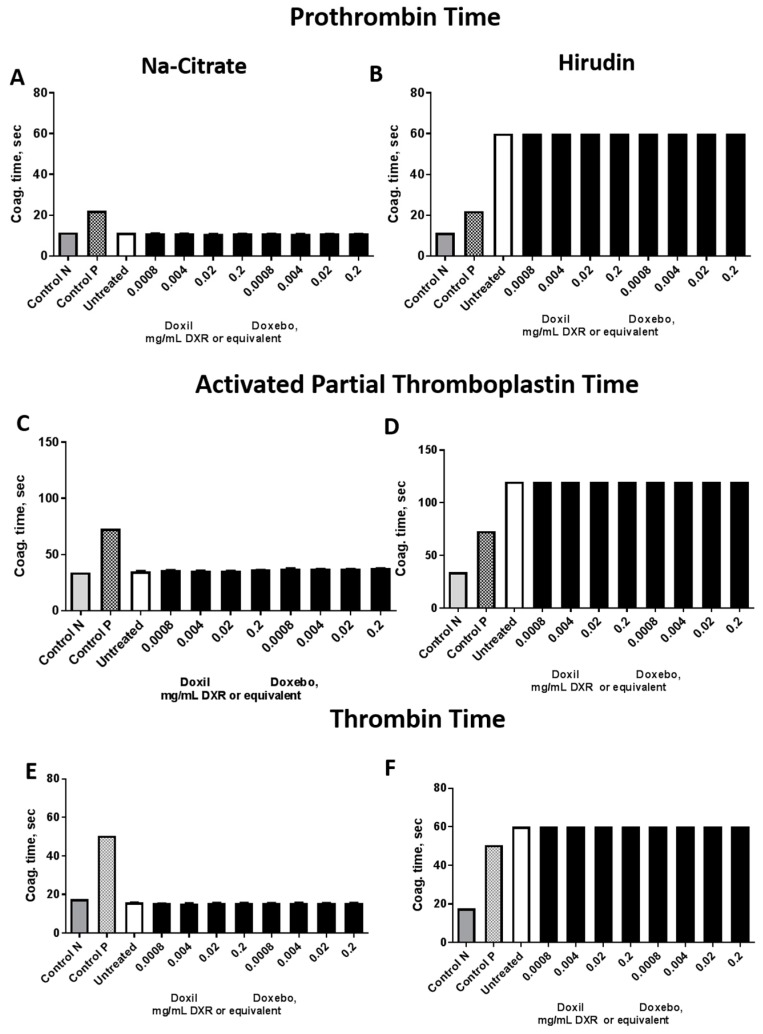
Plasma coagulation time. Various clinically relevant concentrations of Doxil and Doxebo were tested in prothrombin time, thrombin time, and activated partial thromboplastin time assays. For each nanoparticle concentration, three independent samples were prepared and analyzed in duplicate (%CV < 5). Each bar represents the mean (*n* = 3) ± SD. Normal plasma standard (Control N) and abnormal plasma standard (Control P) were used for instrument controls. Plasma pooled from at least three donors was either untreated (Untreated) or treated with nanoparticles at the concentrations shown. Prothrombin time assay in Na-Citrate (**A**) and hirudin (**B**) anticoagulated plasma; Activated Partial Thromboplastin Time in Na-citrate (**C**) and hirudin (**D**) anticoagulated; Thrombin time assay in Na-citrate (**E**) and hirudin (**F**) anticoagulated plasma.

It is common to supplement safety studies that focus on the thrombogenic properties of nanomaterials with an assessment of the effects of nanoparticles on the plasma coagulation time. There are three assays that are commonly used: the prothrombin time (PT) assay, the activated partial thromboplastin time (APTT) assay, and the thrombin time (TT) assay. They allow us to get insight into the functionality of extrinsic, intrinsic, and common pathways, respectively. Normal plasma coagulation time in these assays may be affected by nanoparticle binding to and inhibition of the plasma coagulation factors. Neither Doxil nor Doxebo is known to affect plasma coagulation time [17]. When plasma coagulation assays were conducted in plasma derived from blood anticoagulated with a traditional anticoagulant, sodium citrate, the assay performance was acceptable. The untreated plasma, Control N, and Control ABN were all within the acceptable coagulation time. Neither Doxil nor Doxebo resulted in a change in the plasma coagulation as compared to the untreated plasma control. The results were consistent between PT, APTT, and TT assays (Figure 4). However, when hirudin-anticoagulated blood was used for the experiment, the assay performance was unacceptable as evidenced by the prolongation of plasma coagulation in the untreated plasma sample above levels acceptable for these in vitro assays. Since hirudin inhibits thrombin specifically and irreversibly, and thrombin is a critical component in all of these assays, the inhibition of the coagulation cascade experienced in our study is likely due to the inhibition of the thrombin, which could not be overcome even by the addition of excess thrombin in the TT assay [23]. In the case of sodium citrate, the ability of plasma to coagulate in response to physiological agonists was easily restored by supplying calcium chloride reagent along with the agonists commonly used in all three assays: Neoplastin reagent, CaCl_2_ and thrombin in PT, and APTT and TT assay, respectively. Collectively, this data suggests that hirudin is not an optimal anticoagulant for in vitro plasma coagulation time assessment.

### 2.5. Complement Activation

**Figure 5:**
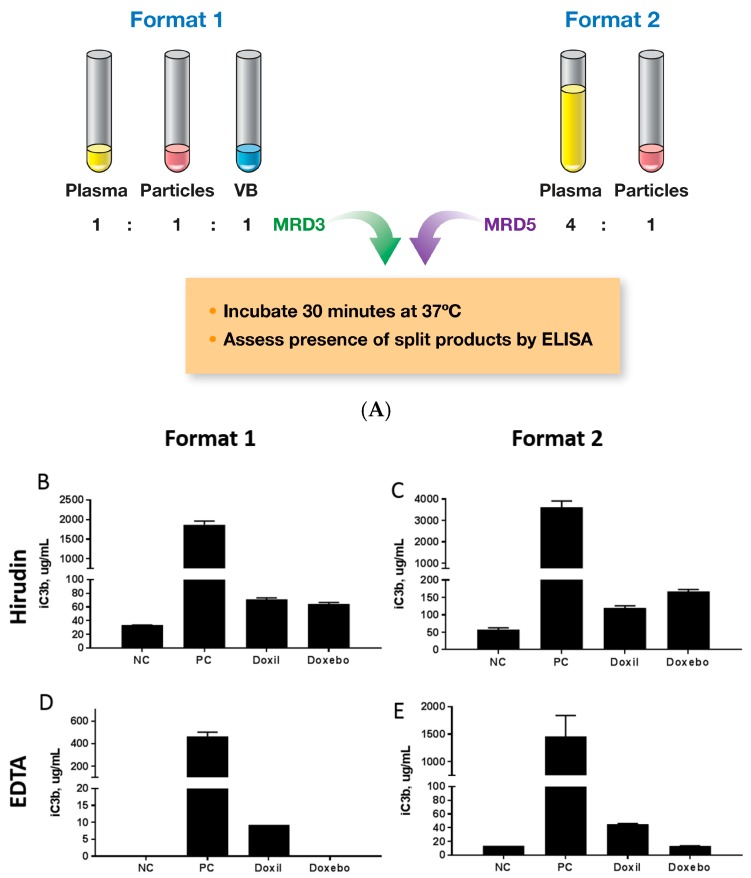
Complement activation. Various clinically relevant concentrations of Doxil and Doxebo were tested in vitro to estimate their effects on the complement system. PBS was used as the negative control (NC). Cobra venom factor (CVF) was used as the positive control (PC). Three independent samples were prepared for each concentration and analyzed in duplicate (%CV < 20). Particle concentration was 0.67 mg/mL of doxorubicin or equivalent for Doxil or Doxebo, respectively. Shown is the mean response (*n* = 3) ± SD. (**A**) Schematic of two assay formats used in this experiment; The results of the assay format 1 using hirudin and EDTA anticoagulants (**B**,**D**, respectively); The results of the assay format 2 using hirudin- and EDTA-anticoagulants (**C**,**E**, respectively). BLOQ = below limit of quantification; VB = veronal buffer; MRD = minimum required dilution.

The complement system is a group of proteins present in human plasma. These proteins represent an innate arm of the immune response and complement the antibody-mediated immune defense mechanism. Three major pathways of the complement activation are known, and they converge on the complement component C3 [24,25,26]. As such, regardless of the mechanism, activation of the complement system can be monitored by analyzing the presence of C3 split products in the test plasma or serum. Desirable complement activation is known to benefit vaccine efficacy, while unwanted complement leads to a condition called complement activation-related pseudoallergy (CARPA) syndrome [24,25,26]. This condition is commonly responsible for the infusion reactions to certain nanomedicines, such as PEGylated liposomal doxorubicin [24,25,26]. There is no universal assay to assess complement activation by nanomedicine. Nevertheless, two formats are commonly used to assess the complement activation in vitro (Figure 5A). We used both of these formats and compared the traditional anticoagulant EDTA to hirudin. Both Doxil and Doxebo induced detectable C3 split product in hirudin anticoagulated plasma (Figure 5B,C). The data was consistent between the assay formats. Similarly, regardless of the assay format, complement activation by Doxil but not by Doxebo was observed in the EDTA anticoagulated plasma (Figure 5D,E). The fact that we detected the complement activation in the EDTA-anticoagulated plasma only with Doxil, but not Doxebo, is consistent with the earlier report, suggesting that a particle surface-exposed or extravesicular doxorubicin can promote complement activation by the liposomes [25]. Our data suggests that this supplementary activation may be insensitive to EDTA, thus, it may proceed via the alternative pathway, which is independent of the presence of C^2+^ ions chelated by the EDTA anticoagulant. Collectively, these data suggest that hirudin may allow for the detection of weaker complement agonists, making it more optimal for the in vitro analysis of nanoparticle effects on the complement system. This finding is in agreement with earlier reports [8,21,24,25,26,27,28].

### 2.6. Leukocyte Proliferation

**Figure 6:**
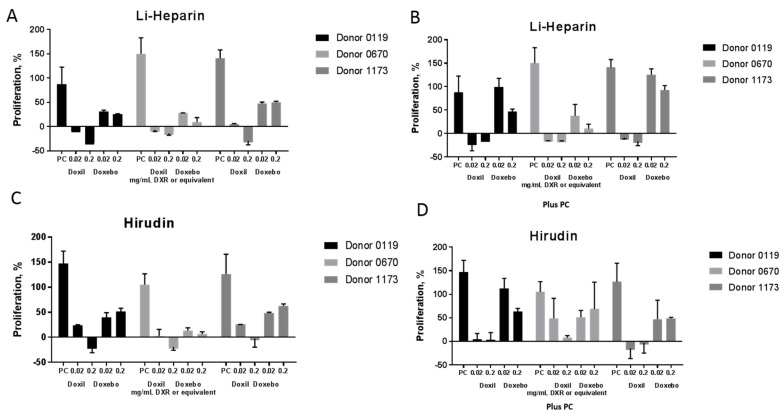
Leukocyte Proliferation PBMC from three healthy donor volunteers was cultured in the presence of nanoparticles and controls for 72 h. Several clinically relevant concentrations of nanoparticles were tested. Following incubation, the proliferation of leukocytes was estimated using the MTT reagent. The percent proliferation was calculated by comparing the mean optical density of test samples to that of the baseline. PBS was used at the negative control (NC). Mitogen phytohemagglutinin (PHA-M) at a concentration of 10 µg/mL was used as the positive control (PC). The experiment included two parts. In part one, the particle’s ability to induce leukocyte proliferation was studied. This part is shown in graphs (**A**,**C**) as Doxil or Doxebo treatments alone. In part two, the ability of nanoparticles to influence mitogen-induced proliferation was assessed. This part is shown in graphs (**B**,**D**) as Doxil or Doxebo plus PC. The data shown in graphs (**A**,**B**) were generated using blood coagulated with Li-heparin. The data shown in graphs (**C**,**D**) were produced using blood anticoagulated with hirudin. Three independent samples were prepared for each treatment and analyzed in duplicate. Percent CV between individual replicates was less than 25. Shown is the mean response ± SD (*n* = 3).

Lymphocytes are a type of white blood cell present in blood. Two main subsets of lymphocytes are B and T cells. When these cells get activated by either a mitogen or an antigen, they proliferate. Understanding the proliferative responses of these cells, therefore, can answer two questions: first, whether a test substance is stimulatory (i.e., whether it acts as a mitogen or as an antigen), and second, whether a test substance is immunosuppressive (i.e., whether it affects lymphocyte proliferation in response to a mitogen or antigen to which the healthy cell would respond under normal conditions). Answering these questions is a very common approach in immunotoxicology when assessing immune cell function. It has also demonstrated fair in vitro and in vivo correlation [19]. We isolated peripheral blood mononuclear cells (PBMC) from human blood anticoagulated with either Li-heparin or hirudin using Ficoll-Paque Plus solution. The isolated cells were then incubated with or without phytohemagglutinin in the presence or absence of nanoparticles and analyzed spectrophotometrically via the MTT (3-(4,5-dimethyl-2-thiazolyl)-2,5-diphenyl-2*H*-tetrazolium bromide) assay. The MTT is processed by metabolically active cells. The increase in the MTT signal corresponds to the increase in the number of viable cells which, in this protocol, is used to assess lymphocyte proliferation. Doxebo did not induce robust lymphocyte proliferation, and the results were consistent between cultures isolated from either heparinized or hirudin-anticoagulated blood (Figure 6A,C). Doxil was toxic to lymphocytes, and it suppressed background proliferation as evidenced by a proliferation percent below that of the untreated cells. This toxicity is not unexpected because Doxil contains the cytotoxic oncology drug doxorubicin, which is known to suppress the proliferation of the dividing cells. Despite the similarity in trends, it appears that cells in cultures obtained from hirudin-anticoagulated blood tolerate the toxicity of Doxorubicin better. This is evidenced by the difference in proliferative responses at a concentration of 0.02 mg/mL between cultures isolated from heparin and hirudin anticoagulated blood (Figure 6A,C). In the second portion of the test, when lymphocyte proliferation induced by a mitogen phytohemagglutinin (PHA-M) was studied, Doxebo was not immunosuppressive, while Doxil suppressed PHA-M-induced proliferation (Figure 6B,D) as expected. Similar to the results in the first phase of the test (compare the data in Figure 6A with that of Figure 6C), cells in cultures isolated from hirudin-anticoagulated blood better tolerated the toxicity of Doxil (compare the data in Figure 6B with that of Figure 6D). This finding is consistent with earlier reports demonstrating that hirudin is friendlier to the blood cells than heparin and other traditional anticoagulants [4,11,29].

### 2.7. Cytokine Response

**Figure 7:**
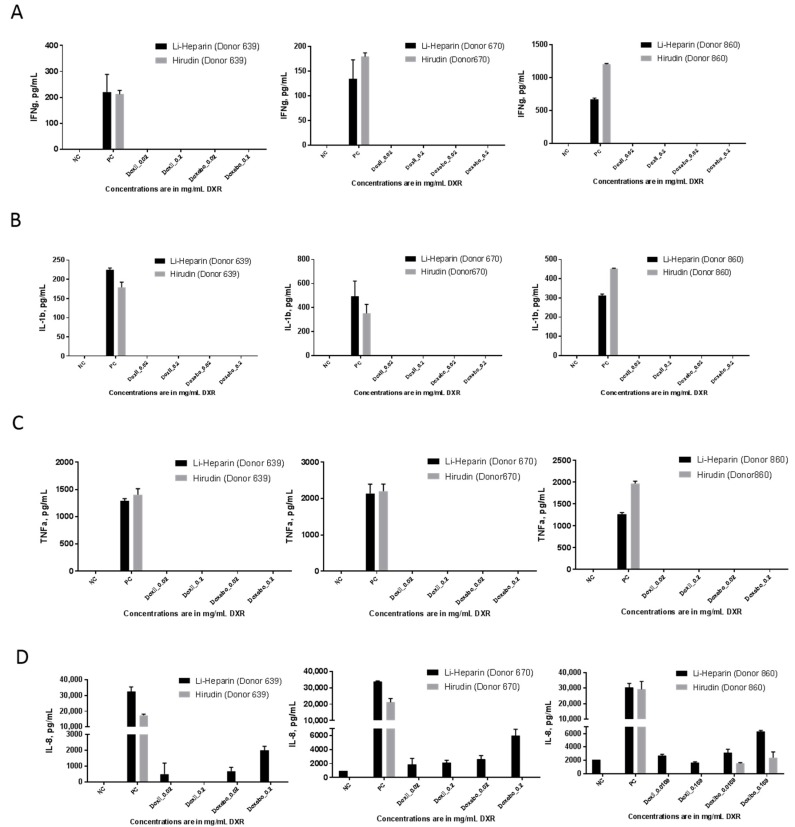
Cytokine response in PBMC. Doxebo and Doxil were tested at two clinically relevant concentrations in the PBMC cultures derived from the blood of three healthy donor volunteers. Donor number is shown in parentheses. PBS was used as the negative control (NC), 10 ng/mL *E. coli* K12 LPS and 10 μg/mL PHA-M were used as PC. Supernatants were analyzed by ELISA to estimate concentrations of IFNγ (**A**); IL1-β (**B**); TNFα (**C**); and IL-8 (**D**). Three independent samples were prepared for each concentration and analyzed in duplicate (%CV < 20). Shown is the mean response (*n* = 3) ± SD. BLOQ = below the limit of quantification.

Cytokines are the biomarkers of inflammation. A cytokine storm, fever, and fever-like reactions are common toxicities limiting translation of certain types of pharmaceuticals and some nanomedicines [19]. In vitro assays using PBMC cultures demonstrated excellent in vitro and in vivo correlation [19]. We isolated PBMC from blood anticoagulated with lithium-heparin or hirudin and compared the induction of several cytokines indicative of the cytokine storm reactions (Figure 7). Assay interference was not detected in either matrix. No difference in the interferon gamma (IFNγ) assay results was seen between cells derived from heparin and hirudin anticoagulated blood. In one donor (RDP860), the positive control response in PBMC from hirudin-anticoagulated blood was stronger than that in the heparin-anticoagulated specimen (Figure 7A). In the interleukin 1 beta (IL-1β) and tumor necrosis factor alpha (TNFα) assays, no difference was seen in PBMC responses to Doxil and Doxebo between cells derived from heparin and hirudin anticoagulated blood (Figure 7B,C). Similar to the type II interferon (IFNγ) results, we found that in the TNFα assay the positive control response in PBMC from hirudin-anticoagulated blood of one donor (RDP860) was stronger than that in cells derived from the heparin-anticoagulated specimen (Figure 7C). In all of these tests, neither Doxil nor Doxebo resulted in a positive cytokine response (Figure 7A–C). We have reported earlier that the pro-inflammatory chemokine interleukin-8 (IL-8) is commonly induced by lipid-based nanoparticles such as liposomes [19]. As expected, Doxebo and, to a lesser extent, Doxil resulted in the induction of this chemokine in our study. However, despite the comparable performance of the positive control assay, Doxil- and Doxebo-mediated induction of IL-8 was consistent only in PBMC derived from Li-heparin-anticoagulated blood (Figure 7D). Cells from the hirudin-anticoagulated blood did not respond to Doxil in all donors and responded to Doxebo only in one donor (Figure 7D). The anticoagulant was washed away during PBMC separation. Therefore, the observed difference in test results is not due to the interaction between anticoagulant and particles. It is likely due to some effects that either hirudin or unidentified impurities in hirudin preparation have on the blood cells at the time of blood collection and transportation to the lab. The nature of such changes is unknown at this time. Another important finding from this study is that cytokine response to the traditional immunological control LPS is not affected. The data suggest that proinflammatory properties of a weaker stimulus, such as liposomes, may be overlooked if hirudin is used as an anticoagulant. It is also not improbable that other nanoparticles with weak pro-inflammatory properties may perform differently in this test when hirudin is used as an anticoagulant. Altogether, the data advise that the evaluation of various anticoagulants may be needed for each nanoparticle formulation in order to select the conditions most optimal for the given nanoparticle. Relying solely on the traditional immunological controls (e.g., LPS) may lead to the selection of suboptimal conditions and, therefore, increase the chance of overlooking the pro-inflammatory property of a given nanoformulation.

## 3. Materials and Methods 

### 3.1. Reagents

Hirudin blood collection Monovette tubes were purchased from Sarstedt Corporation (Newton, NC, USA) under a Material and Transfer Agreement. Lithium heparin, sodium citrate, and EDTA vacutainers were purchased from BD Biosciences (San Jose, CA, USA). Collagen was purchased from ChronoLog (Havertown, PA, USA). PT, APTT, and thrombin time assay reagents were obtained from Diagnostica Stago (Parsippany, NJ, USA). Roswell Park Memorial Institute (RPMI), fetal bovine serum (FBS), pen/strep, Dulbecco’s Phosphate Buffered Saline (DPBS) (Ca^2+^/Mg^2+^ free), and Hank’s Balanced Salt Solution (HBSS) were purchased from Thermo Biosciences Holdings LLC (Waltham, MA, USA). LPS was from Invivogen (San Diego, CA, USA). PHA-M and MTT were from Sigma-Aldrich (St. Louis, MO, USA). Hemoglobin standard and CMH reagents for the hemolysis assay were from StanBio (Boerne, TX, USA) and Teco Diagnostics (Anahem, CA, USA), respectively. Antibodies, standards, and conjugates for cytokine assays were from R&D Systems (Minneapolis, MN, USA). Doxil and Doxebo were purchased from Avanti Polar Lipids (Alabaster, AL, USA). According to the material certificate of analysis, the concentration of the anticancer drug doxorubicin was 2 mg/mL in the Doxil formulation. Both Doxil and Doxebo had comparable total lipid concentrations. For in vitro assays, Doxil was diluted to final concentrations of 0.2, 0.02, 0.004, and 0.008 mg/mL of doxorubicin. Doxebo was analyzed at total lipid concentrations identical to that in the Doxil formulation when it contained 0.2–0.008 mg/mL of doxorubicin. For simplicity, we refer to these concentrations as that of doxorubicin in the case of Doxil or doxorubicin equivalent in the case of Doxebo. The two lowest concentrations (0.0008 and 0.004 mg/mL) were skipped in the hemolysis and cell-based assays as toxicity was not expected at these concentrations, as well as to avoid the logistical challenges of processing multiple samples side-by-side. Platelet aggregation and plasma coagulation assays assessed a full range from 0.0008 to 0.2 mg/mL. For the complement activation assay, the tested final concentration was 0.6 mg/mL, which is the highest achievable in vitro concentration. It was used to mimic the conditions of the infusion reactions caused by a high local concentration at the time of injection.

### 3.2. DLS and Zeta Potential

A Malvern Zetasizer Nano ZS instrument (Southborough, MA, USA) with back scattering detector (173°, 633 nm laser wavelength) was used for measuring the hydrodynamic size (diameter) in batch mode at 25 °C in a low volume quartz cuvette (pathlength 10 mm). Stock liposome samples were diluted 100-fold in PBS before a minimum of twelve measurements were made. Hydrodynamic size was reported as the intensity-weighted average (Int-Peak). A Malvern Zetasizer Nano ZS instrument was used to measure zeta potential at 25 °C. Stock liposome samples were diluted 100-fold in 10 mM NaCl and their pH measured (pH 6.6). An applied voltage of 150 V was used. Samples were loaded into pre-rinsed folded capillary cells and a minimum of three measurements were made per sample.

### 3.3. Research Donor Blood

Healthy volunteer blood specimens were drawn under National Cancer Institute (NCI) at Frederick Protocol OH99-C-N046. Blood was collected in BD vacutainer tubes containing sodium citrate, lithium heparin, EDTA, or hirudin as an anticoagulant. The blood was collected from different donors, however, for the purpose of the comparative study, hirudin and traditional anticoagulants were used to collect the blood from the same three donors for each assay.

### 3.4. Endotoxin

To study potential particle contamination with endotoxin, the test samples were analyzed by turbidity Limulus Amoebocyte Lysate (LAL) assay according to the protocol at http://ncl.cancer.gov/NCL_Method_STE-1.2.pdf [30,31]. No endotoxin was detected in any test sample at concentrations used in the in vitro assays.

### 3.5. Hemolysis

An analysis of nanoparticle hemolytic properties was conducted using Nanotechnology Characterization Lab (NCL) protocol immunotoxicity assay (ITA-1), (https://ncl.cancer.gov/sites/default/files/protocols/NCL_Method_ITA-1.pdf) [32]. In brief, freshly drawn human blood anticoagulated with either lithium heparin or hirudin was diluted in PBS to a concentration of 10 mg/mL total blood hemoglobin. The diluted whole blood was then incubated with test samples for three hours at 37 °C. Following incubation, cell-free supernatants were prepared and analyzed for the presence of plasma-free hemoglobin by converting hemoglobin and its metabolites into cyanmethemoglobin (CMH) using Drabkin’s reagent. CMH was then quantified against a hemoglobin standard by measuring the absorbance of the samples at 540 nm. Triton X-100 was used as the assay positive control.

### 3.6. Platelet Aggregation

Particle effects on platelet aggregation were analyzed using NCL protocol ITA-2.2 (http://ncl.cancer.gov/NCL_Method_ITA-2.2pdf) [33]. Briefly, whole blood anticoagulated either with sodium citrate or hirudin was centrifuged for eight minutes at 200× *g* to obtain platelet-rich plasma (PRP). The PRP was treated with nanoparticles, saline (negative control), or collagen (positive control), and platelet aggregation was monitored in real time for six minutes at 37 °C using a light transmission aggregometer, Chrono-Log Model 700 (Chronolog corporation, Havertown, PA, USA). To test the particles’ effects on the collagen-induced aggregation, the PRP was pre-treated with test nanoparticles, and aggregation was then induced using collagen.

### 3.7. Complement Activation

Two procedures were followed. In assay format one, the experimental procedure described in ITA-5.2 (https://ncl.cancer.gov/sites/default/files/protocols/NCL_Method_ITA-5.2.pdf) was followed [34]. In assay format two, the procedure described by Dr. Szebeni [21,25] was followed. Briefly, plasma was prepared from freshly drawn human blood anticoagulated with either EDTA or hirudin. Plasma from three donors was pooled and either incubated with test samples and veronal buffer at the assay minimum required dilution of three, or with test samples at the assay minimum required dilution of five for 30 min at 37 °C. Following incubation, the samples were analyzed for the presence of the iC3b component of the complement using a commercial enzyme immunoassay kit. The schematic representation of the assay formats is shown in Figure 5. Cobra venom factor (CVF) was used as a positive control in this assay.

### 3.8. Plasma Coagulation Time

We used NCL protocol ITA-12 (https://ncl.cancer.gov/sites/default/files/protocols/NCL_Method_ITA-12.pdf) [33]. Briefly, three plasma coagulation tests were performed: prothrombin time (PT), activated partial thromboplastin time (APTT), and thrombin time (TT). Freshly drawn human blood anticoagulated with either sodium citrate or hirudin was used to prepare the plasma. Plasma from three donors was pooled and incubated with test samples for 30 min at 37 °C. Following incubation, plasma coagulation initiation reagents (neoplastin, CaCl_2_, or thrombin, respectively) were added to the mixture, and the coagulation times were measured using the STArt4 coagulometer (Diagnostica Stago, Parsippany, NJ, USA).

### 3.9. Leukocyte Proliferation

Experiments were performed according to NCL protocol ITA-6 (https://ncl.cancer.gov/sites/default/files/protocols/NCL_Method_ITA-6.pdf) [35]. Briefly, whole blood anticoagulated with either lithium heparin or hirudin was diluted in PBS, and PBMC were isolated using the Ficoll Paque gradient density centrifugation. Purified PBMC were incubated with controls and nanoparticle samples for 72 h. At the end of incubation, a cell number indicative of cell proliferation was determined using the MTT assay. PHA-M was used as a positive control in this assay.

### 3.10. Cytokine Response in PBMC Cultures

Experiments were performed according to NCL protocol ITA-10 (https://ncl.cancer.gov/sites/default/files/protocols/NCL_Method_ITA-10.pdf) [36]. Briefly, whole blood anticoagulated with either lithium heparin or hirudin was diluted in PBS, and PBMC were isolated using Ficoll Paque gradient density centrifugation. Purified PBMC were incubated with controls and nanoparticle samples for 24 h. 10 ng/mL *E. coli* K12 LPS and 10 μg/mL PHA-M were used as positive controls in this assay. At the end of incubation, the samples were centrifuged for five minutes at 18,000× *g*, and supernatants were analyzed for the presence of pro-inflammatory cytokines (IL-8, IL-1β, and TNFα) and type II interferon (IFNγ) using NCL protocols ITA-22, ITA-23, ITA-24, and ITA-25, respectively [36]. (https://ncl.cancer.gov/sites/default/files/protocols/NCL_Method_ITA-22.pdf; https://ncl.cancer.gov/sites/default/files/protocols/NCL_Method_ITA-23.pdf; https://ncl.cancer.gov/sites/default/files/protocols/NCL_Method_ITA-24.pdf; https://ncl.cancer.gov/sites/default/files/protocols/NCL_Method_ITA-25.pdf).

## 4. Conclusions

Overall, the results of our study are consistent with several earlier reports and suggest that hirudin may be more optimal for some in vitro assays but less ideal for others. Specifically, hirudin demonstrated comparable performance to traditional anticoagulants in hemolysis and leukocyte proliferation assays (Table 2). Unlike traditional anticoagulants, hirudin allows for the detection of the weaker stimulus in the complement activation assay. Therefore, it should be considered as the most optimal anticoagulant for the in vitro analysis of nanoparticle effects on the complement system. In contrast to traditional anticoagulants, hirudin was not optimal for the plasma coagulation, platelet aggregation, and cytokine secretion assays (Table 2). Most importantly, we found that relying solely on the traditional immunological controls (e.g., LPS) may lead to the selection of suboptimal conditions in the cytokine secretion assay and increase the chance of overlooking the pro-inflammatory property of the given nanoformulation, especially when such a formulation is a less potent cytokine inducer than the assay positive control. Our data further emphasize the importance of recognizing the differences in test results based on the type of anticoagulant used to prepare blood. It is, therefore, critical to consider anticoagulants both during assay validation and interpretation of the study results.

## Figures and Tables

**Table 1:** Particle description and characterization summary. Each nanoparticle was tested at several dilutions. Endotoxin levels reported in this table are from the dilution 1:50. ** Doxebo interfered with Limulus Amoebocyte Lysate (LAL) at dilution 1:5, therefore no valid data can be obtained from that dilution; * Doxil did not interfere with the LAL at the dilution 1:5, and the result at that dilution was 0.08 EU/mL; DXR = doxorubicin.

Model Particles	Hydrodynamic Size (nm)	Zeta Potential (mV)	Drug (Conc. in mg/mL)	Endotoxin, EU/mL
Doxil	87.8	−4.0	DXR (2.0)	<0.5 *
Doxebo	83.1	−4.0	None	<0.5 **

**Table 2:** Recommendation for anticoagulant use in in vitro assays for nanoparticle characterization. Each anticoagulant has its own mechanism of action. The type of anticoagulant may influence assay performance. Each anticoagulant in this table matches the assay for which it provided the optimal assay performance. Assays noted with a * mean that while this anticoagulant is not ideal for the given assay, it can be used in the situations where hirudin is not available. It should also be noted that EDTA is commonly used in clinic if the blood analysis requires an estimation of the platelets count. MOA: mechanism of action.

Anticoagulant	Main MOA	Targets outside of the Blood Coagulation	Assay Recommendation
EDTA	Chelation of divalent cations	yes	Complement activation *
Citrate	Chelation of divalent cations	yes	Platelet aggregation, Plasma Coagulation
Heparin	Inhibition of intrinsic, extrinsic and common coagulation pathways	yes	Hemolysis, Cytokines, Leukocyte proliferation *
Hirudin	Specific Thrombin inhibition	no	Complement activation, Hemolysis, Leukocyte Proliferation
